# Transfer Learning from Homogeneous to Heterogeneous: Fine-Tuning a Pretrained Interatomic Potential for Multicomponent Mo Alloys with Localized Substitutional Alloying

**DOI:** 10.3390/ma19091715

**Published:** 2026-04-23

**Authors:** Lixin Fang, Liqin Qin, Limin Zhang, Hao Zhou, Xudong He, Zekun Ren, Tongyi Zhang, Yi Liu

**Affiliations:** 1 Materials Genome Institute, Shanghai University, Shanghai 200444, China; 2 State Key Laboratory of Materials for Advanced Nuclear Energy, Shanghai University, Shanghai 200444, China; 3 Berkeley Education Alliance for Research in Singapore (BEARS), Create Tower, 1 Create Way, Singapore 138602, Singapore; 4 Institute of Low-Dimensional Carbons and Device Physics, Shanghai University, Shanghai 200444, China

**Keywords:** machine learning interatomic potentials (MLIPs), MACE foundation model, transfer fine-tuning, localized substitutional doping, multicomponent Mo alloy design

## Abstract

Machine learning interatomic potentials (MLIPs) are typically developed for globally ordered homogeneous systems (GOHomS), which exhibit only minor local deviations from equilibrium configurations. Consequently, most existing MLIPs trained on GOHomS often perform inadequately when applied to locally ordered heterogeneous systems (LOHetS), e.g., substitutional alloying elements in multicomponent alloys. To describe doping alloy systems, we develop a fine-tuned MLIP based on the MACE foundation model, specifically tailored for Mo-based dilute alloys containing one or two out of 20 substitutional elements: Cr, Fe, Mn, Nb, Re, Ta, Ti, V, W, Y, Zr, Al, Zn, Cu, Ag, Au, Hg, Co, Ni, and Hf. The model is built on more than 7000 equilibrium and non-equilibrium structures derived from first-principles density functional theory (DFT) calculations. The optimized large-scale fine-tuned model attains state-of-the-art accuracy, with a mean absolute error (MAE) and root-mean-square error (RMSE) of 2.27 meV/atom and 3.79 meV/atom for energy predictions, and 13.83 meV/Å and 24.26 meV/Å for force predictions, respectively. Systematic evaluation under different data-splitting protocols shows that unknown element extrapolation remains challenging under strict dopant hold-out, whereas substantially improved accuracy can be achieved in partial-exposure transfer settings. The fine-tuned models reduce the MAE by approximately 7–10 times compared to models trained from scratch, and by 10–20 times relative to zero-shot foundation models. This performance gain remains consistent across varying dataset sizes (equilibrium vs. non-equilibrium structures) and model scales. Our work illustrates the efficacy of transfer learning from globally ordered homogeneous systems to locally ordered heterogeneous multicomponent alloy environments. However, direct transfer to entirely unknown elements remains challenging, especially when proxy embeddings are employed without fine-tuning. Thus, to achieve high accuracy without incurring additional cost, it is essential to include unknown elements in the training dataset while minimizing the number of configurations containing known elements. Moreover, the current findings are primarily validated for dilute Mo-based alloy systems. Extending this approach to more compositionally complex alloy spaces may necessitate additional data and further fine-tuning.

## 1. Introduction

Locally ordered heterogeneous systems (LOHetS) represent a key category of materials science concepts, encompassing substitutional or interstitial doping, defect configurations (e.g., vacancies, stacking faults, twins, dislocations, grain boundaries), and chemical short-to-medium-range order in high/medium-entropy materials. These systems are characterized by local variations in chemistry—such as element type, concentration, arrangement, and site occupancy—or in structural features, including substitutional versus interstitial occupation. Beyond serving as distinct structural entities, LOHetS can also act as building blocks for statistically hierarchical and ensemble-based bulk materials, thereby providing a critical foundation for understanding macroscopic material properties at the atomic level. In contrast, machine learning interatomic potentials (MLIPs) are typically developed for globally ordered homogeneous systems (GOHomS), which exhibit only minor local deviations from equilibrium configurations. Consequently, most existing MLIPs trained on GOHomS often perform inadequately when applied to LOHetS. Addressing this challenge constitutes the primary motivation of the present work.

MLIPs are increasingly developed to bridge first-principles accuracy and large-scale simulation efficiency via data-driven implicit energy expressions of a deep learning model [1,2,3,4,5]. MLIPs have evolved from descriptor-based and kernel approaches (BPNN, GAP, SOAP, SNAP, MTP, ACE) [6,7,8,9,10,11] to end-to-end graph and equivariant neural networks (SchNet, DimeNet, GemNet, PaiNN, NequIP, MACE) [12,13,14,15,16,17]. State-of-the-art performance of MLIPs is typically driven by large-scale pretraining and reported as low energy/force errors on benchmark datasets [18,19,20,21,22,23,24,25]. Although model capacity and benchmark accuracy have advanced substantially, robustness to distribution shift has not progressed at the same pace. High in-domain accuracy does not necessarily translate into stable extrapolation in task-specific material settings [26,27,28], which hinders their practical applications in alloy design.

With the emergence of foundation models, pretraining followed by downstream adaptation has become the dominant workflow. Evidence from M3GNet, CHGNet, MACE-MP-0, and MatterSim indicates that universal pretraining can markedly reduce downstream data requirements [18,19]. Frozen-transfer and partial fine-tuning studies further report measurable gains in low-data regimes [23,29,30,31]. At the same time, benchmark suites such as OC20/OC22 and Matbench underscore the need for unified out-of-distribution (OOD) evaluation protocols [26,27]. Recent studies have further emphasized that the reliability of MLIPs depends not only on model architecture, but also critically on the coverage, diversity, and physical relevance of the training data [32,33]. Moreover, low benchmark errors do not necessarily imply robust generalization under OOD conditions, especially in chemically complex or high-energy environments [34,35]. However, many existing benchmarks usually involve limited train–test distribution shift, with data dominated by perfect crystals or near-equilibrium globally deformed structures and relatively limited local-environment diversity [36,37,38]. By contrast, substitutional alloy systems involve coupled variations in local composition, site occupancy, bonding topology, and geometric distortion. These locally heterogeneous environments play important roles in real engineering alloys and may be more complex than global structures. Recent studies [39,40] further suggest that local chemical ordering and dopant-dependent environments can influence MLIP accuracy and transferability. In this context, the present Mo-based study provides a useful case for examining transfer learning from globally ordered homogeneous crystal systems to locally ordered heterogeneous alloy environments. In multicomponent alloy design, e.g., substitutionally doped systems, the local heterogeneous characteristics differs from the globally uniform crystals. Doping simultaneously perturbs elemental composition, site occupancy, and local bonding, causing coupled shifts in both structural and chemical distributions. When held-out dopants are absent from training data, prediction becomes strict OOD extrapolation rather than interpolation. In our work, OOD refers to two related but distinct forms of distribution. The first is the transferability from globally ordered homogeneous to locally ordered heterogeneous environments in the Mo-based systems. The second is element-level extrapolation, where the model is applied to chemical elements that are not included in the original training and fine-tuning datasets. The central question is therefore whether a foundation model pretrained on global crystal data can, after task-specific fine-tuning, deliver stable and reproducible joint energy-force predictions on locally doped systems.

Mo-based alloys are representative materials for extreme-service high-temperature environments because of their high melting point, elevated-temperature strength, and irradiation resistance. Since a large number of alloying elements can be doped into the conventional BCC and hypothetical FCC host lattices, the dopant-composition space expands rapidly, whereas exhaustive enumeration of first-principles density functional theory (DFT) calculations remains computationally expensive for iterative design [41,42,43,44,45]. A systematic assessment of the transfer learning workflow via fine-tuning a pretrained foundation model on locally doped data is therefore of direct scientific and engineering relevance.

This work aims to examine the transferability of pretrained foundation model, e.g., MACE (Message Passing Atomic Cluster Expansion) in substitutional-doping tasks. Under unified data organization and evaluation criteria, we compare zero-shot, scratch, and fine-tune settings to address two questions: (i) whether fine-tuning materially improves predictive fidelity for doped systems, and (ii) whether these gains remain stable under leave-one-dopant-out cross-element evaluation.

To address these questions, we establish a unified validation pipeline on a Mo substitutional-doping dataset in which each structure has both per-atom energy and atomic force labels. We compare zero-shot, scratch, and fine-tuning paradigms under matched settings, and evaluate both in-distribution fitting and cross element transfer. Detailed descriptions of the dataset partitioning and evaluation protocols, are provided in Section 2. We further perform direct inference on elements absent from training to probe practical deployment limits for unseen dopants. This boundary test uses proxy-element mapping (e.g., an unseen element may be temporarily assigned to a chemically related seen element such as Hf to Zr for inference) and should not be interpreted as strict modeling of true unseen chemical identities.

The contributions in this study are threefold. First, we establish standardized data organization and evaluation protocols for Mo-doping systems, together with a reproducible joint energy-force training workflow. Second, we quantify transfer gains from global crystal pretraining to locally doped distribution adaptation through direct comparison of zero-shot, scratch, and fine-tune paradigms. Third, we characterize both effective opening regions and failure boundaries of fine-tuning under independent and identically distributed (IID) and dopant-level leave-one-dopant-out (LODO) protocols, providing practical guidance for re-training and rapid calibration on new dopants. Overall, this study demonstrates a transfer learning protocol from globally ordered homogeneous systems to locally ordered heterogenous systems that provides a generally practical tool for multicomponent alloy design. The similar approach can be extended to broad doping systems including multicomponent ceramics and semiconductors, even more general locally ordered heterogeneous systems (LOHetS).

## 2. Methods and Datasets

### 2.1. Fine-Tuning Dataset of Doped Mo Alloys

Before introducing the task-specific Mo-doping dataset, we briefly summarize the pretraining corpus of the base checkpoint used in this work (MACE-MP-0). The MACE-MP family is pretrained on the MPtrj dataset, which aggregates static calculations and ionic-relaxation trajectories from the Materials Project [46]. The architecture of MACE models is shown in Text S1 of the Supplementary Materials (SM). In essence, this pretraining distribution is dominated by ideal or slightly distorted crystalline environments whose chemical constituents are uniformly distributed following a global crystal symmetry, whereas our target dataset focuses on substitutionally doped Mo systems with explicit local chemical-disorder and geometrical-distortion effects. This training data domain gap is exactly why fine-tuning on the heterogenous doped dataset is necessary in this study.

The dataset used in this work was obtained from first-principles (DFT) calculations (VASP, version 6.3.0) of substitutionally doped Mo alloys in both BCC and FCC lattice, using the GGA-PBE [47,48] exchange-correlational functional, the same as that in MACE model construction. Our calculations on the doped Mo alloys take into account the spin polarization that is critical to magnetic elements. The conventional stable structure for Mo is BCC at ambient conditions, while the hypothetical FCC Mo provides more high-energy metastable structures, especially the closet-packed crystal environment for the dopants, which improves the size and diversity of training data. Additional DFT computational details are shown in Text S2 of the Supplementary Materials.

**Figure 1:**
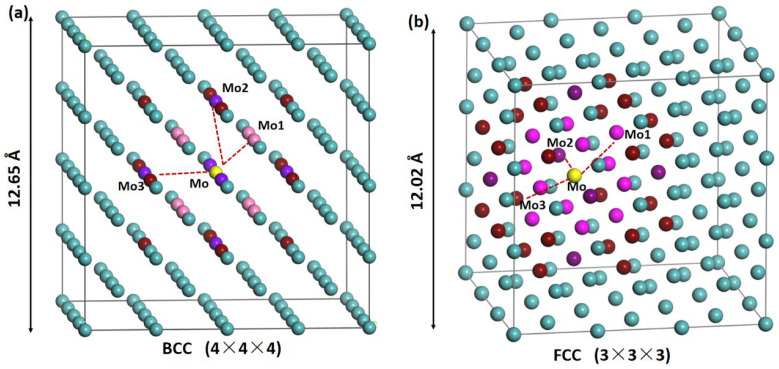
Representative Mo-host supercells used in this work: (**a**) BCC 4 × 4 × 4 and (**b**) FCC 3 × 3 × 3. Cyan spheres denote host-lattice atoms, and the highlighted sites (Mo, Mo1, Mo2, and Mo3) indicate the central reference position and representative substitutional-doping locations. The supercell edge lengths are 12.65 Å (BCC) and 12.02 Å (FCC), respectively.

As shown in Figure 1a,b, the local doping environment is defined around a central reference Mo site together with representative neighboring sites (Mo1–Mo3), which are used to construct substitutional configurations in the BCC and FCC host lattices. Following common Mo-based alloy systems, we studied 11 dopant elements at a dilute concentration (0.78–1.85 at%), including Cr, Fe, Mn, Nb, Re, Ta, Ti, V, W, Y, and Zr, to substitute for Mo1–Mo3 sites. The dataset contains single-site and double-site substitution samples with the first, second, and third neighbors, corresponding to one or two alloying elements out of the 11 candidates in a BCC (128 atoms) or FCC (108 atoms) supercell with fixed lattice parameters of the optimized pure Mo. This choice was made under the dilute doping limit considered in this work (0.78–1.85 at%), where the influence of a single dopant on the global lattice constant is expected to remain limited. In addition, allowing full cell relaxation would change the supercell dimensions and introduce an additional source of variation, as well as possible size-dependent effects, which could complicate the comparison among different doped configurations.

Let D(s) denote the dopant-element set of structure s. |D(s)| = 1 and 2 represent the single-dopant and double-dopant configurations, respectively. This definition is used consistently for data grouping in IID and LODO protocols. For data management and reproducibility, samples are partitioned by configuration state into FT-E (equilibrium/near-equilibrium) and FT-E_NE (equilibrium and non-equilibrium), containing 420 and 6970 structures, respectively. The FT-E_NE dataset contains FT-E samples, so the full dataset has 6970 structures where FT-E provides near-equilibrium structural information and FT-E_NE expands coverage of perturbed local environments during the geometrical relaxation process.

Each data sample retains complete structure–energy–force information:

(1) $$s_{n}=\left(R_{n},Z_{n},e_{n},{\left\{F_{n,i}\right\}}_{i=1}^{M_{n}}\right)$$

where $R_{n}$ is the atomic coordinate matrix, $Z_{n}$ denotes element identities, $e_{n}$ is the per-atom energy, $F_{n,i}$ is the force label of atom i, and $M_{n}$ is the number of atoms in structure n. Energy and force units are standardized as meV/atom and meV/Å, respectively.

All ML model construction was conducted on this unified dataset, excluding the verification datasets of new elements in Section 3.3. Data variability is controlled along three dimensions: matrix crystals (BCC/FCC), dopant combination (single/double site), and configuration state (E/E_NE).

### 2.2. Model Training Protocols and Evaluation Metrics

(1) Model settings

To evaluate the interplay between global pretraining priors and local-domain adaptation, we construct three directly comparable settings (zero-shot, scratch, and fine-tune) under unified data protocols and model scales (Table S1 of the Supplementary Materials). The three training paradigms are described as follows. (a) Zero-shot model: direct inference on the target system using a pretrained MACE checkpoint, without parameter updates, to quantify cross-domain transferability. (b) Scratch model: training from random initialization under the same architecture, to characterize achievable performance without pretrained priors. (c) Fine-tuned model: continued optimization from pretrained weights on target-domain data, to quantify error reduction and adaptation gains from transfer learning. The three settings differ only in parameter initialization and whether weights are updated. All the other training and evaluation conditions are identical.

To investigate the data-size effects of the fine-tuning, we further evaluate two fine-tuning data regimes: FT-E (equilibrium fine-tuning), using 420 equilibrium/near-equilibrium structures (small dataset); FT-E_NE (non-equilibrium fine-tuning), using 6970 non-equilibrium structures (large dataset). These two settings share the same pretrained initialization and training configuration (optimizer, learning-rate schedule, batch organization, and early-stopping strategy) and differ only in fine-tuning data source and scale. FT-E and FT-E_NE are not sample-size matched. Therefore, this comparison is intended for practical performance assessment rather than strict single-factor causal attribution to equilibrium versus non-equilibrium state. Under this unified setup, the FT-E versus FT-E_NE comparison addresses two questions: whether small equilibrium datasets provide stable energy calibration, and whether large non-equilibrium datasets improve local potential-energy-surface coverage and overall error profiles. Quantitative comparison results are reported in Section 3.

(2) Data-splitting and evaluation protocols

Two data-splitting protocols were used in this work: independent and identically distributed (IID) and dopant-level leave-one-dopant-out (LODO). The IID protocol randomly splits all samples into training, validation, and test sets at a (81:9):10 ratio. The random splits were independently performed 10 times for the equilibrium data and five times for the non-equilibrium data. The non-equilibrium dataset is substantially larger than the equilibrium dataset and therefore needs much more cost for repeated training. Under the condition that the model performance had shown stable convergence behavior, five repeated runs were adopted for the non-equilibrium dataset as a practical compromise between computational cost and statistical reliability. This protocol evaluates interpolation performance under matched distributions.

The LODO protocol builds extrapolative tests by grouping data by the type of dopant element. Under LODO, each element fold is trained and tested independently. For any held-out element x, we apply two data partition rules: (a) Strict-LODO: if x∈D(s), sample s is assigned to the test set. For double-site substitution samples, the presence of x at either site is sufficient for the test assignment rather than in the training dataset, which means the new element for the test was completely unseen in the training. This strategy maximally avoids elemental information leakage and provides stricter transfer assessment. (b) Relaxed-LODO: single-site samples are assigned by x∈D(s); double-site samples are assigned to test only when both sites satisfy the hold-out rule while the new element that appeared in one of the double sites was partially seen in the training. This protocol is less restrictive and estimates an upper performance bound under partial element co-occurrence.

We report mean and standard deviation across folds to quantify element-wise stability. Model selection is based on validation performance only; the test set is used once for final reporting. For fair comparison, scratch and fine-tune share identical data splits, graph-construction settings, batch organization, optimizer, and learning-rate schedule.

(3) Loss functions and evaluation metrics

Training jointly constrains energy and atomic forces. Let the dataset contain N structures, where structure n has $M_{n}$ atoms. Predicted and reference per-atom energies are ${\hat{e}}_{n}$ and $e_{n}$; predicted and reference atomic forces are ${\hat{F}}_{n,i}$ and $F_{n,i}$. The energy and force terms are

(2) $$L_{E}=\frac{1}{N}\sum_{n=1}^{N}\left|{\hat{e}}_{n}-e_{n}\right|,\;\;L_{F}=\frac{1}{\sum_{n=1}^{N}M_{n}}\sum_{n=1}^{N}\sum_{i=1}^{M_{n}}{∥{\hat{F}}_{n,i}-F_{n,i}∥}_{2}$$

The total loss is defined as

(3) $$L=\lambda_{E}L_{E}+\lambda_{F}L_{F}$$

The equal weights $\lambda_{E}=\lambda_{F}=30$ were used to calculate the total loss in this work. In the original MACE formulation, the loss terms are combined using predetermined coefficients, with the pretraining setting (λE, λF) = (1, 10), where the force term is emphasized more strongly in the initial stage. In the present work, however, the target dataset contains both equilibrium and non-equilibrium structures, and the forces of the equilibrium subset are generally close to zero. Therefore, rather than directly adopting the original pretraining ratio, we assigned equal importance to energy and force prediction in the fine-tuning task. After preliminary parameter tuning, λE = λF = 30 was adopted in this work as an empirically suitable setting for the present task. This setting balances energy calibration and local potential-energy-surface fidelity, mitigating ranking instability and geometric inconsistency that can arise under single-objective optimization. The <MAE> and <RMSE> averaged over the independent split runs were used as evaluation metrics of ML models for both energy and force whose units are meV/atom and meV/Å, respectively.

Let the test set contain N structures, with predicted and reference per-atom energies ${\hat{e}}_{n}$ and $e_{n}$. The energy metrics are

(4) $${MAE}_{E}=\frac{1}{N}\sum_{n=1}^{N}\left|{\hat{e}}_{n}-e_{n}\right|,\;\;{RMSE}_{E}=\sqrt{\frac{1}{N}\sum_{n=1}^{N}{\left({\hat{e}}_{n}-e_{n}\right)}^{2}}$$

For force metrics, where structure n has $M_{n}$ atoms and predicted/reference forces are ${\hat{F}}_{n,i}$ and $F_{n,i}$, we define

(5) $${MAE}_{F}=\frac{1}{\sum_{n=1}^{N}M_{n}}\sum_{n=1}^{N}\sum_{i=1}^{M_{n}}{∥{\hat{F}}_{n,i}-F_{n,i}∥}_{2}$$

(6) $${RMSE}_{F}=\sqrt{\frac{1}{\sum_{n=1}^{N}M_{n}}\sum_{n=1}^{N}\sum_{i=1}^{M_{n}}{∥{\hat{F}}_{n,i}-F_{n,i}∥}_{2}^{2}}$$

For reproducibility, the key implementation settings for training and inference are briefly summarized here. The energy and force labels are specified as REF_energy and REF_forces, respectively. The learning rate is set to 3 × 10^−4^, and the weight decay is 1 × 10^−6^. The ema_decay is set to 0.99, and the seed is set to 42. Additional implementation details are summarized in Table S1 of the Supplementary Materials.

## 3. Results and Discussion

**Figure 2:**
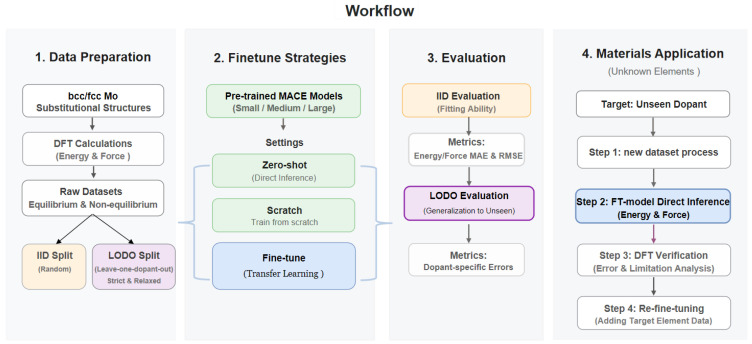
Workflow of fine-tuning the pretrained MACE model for doped Mo alloys compared with scratch models, followed by the verification on the unknown systems with new elements.

In this work the workflow consists of four stages (Figure 2) as follows: (i) preparation and splitting of BCC/FCC doping datasets; (ii) construction of three ML models based on pretrained MACE: zero-shot, scratch, and fine-tune models; (iii) joint energy-force evaluation under IID and LODO data protocols; and (iv) verification and error analysis for unseen dopant elements. The model performances are compared across the zero-shot, scratch, and fine-tune paradigms under IID and LODO data protocols. The evaluation metrics are energy <MAE>/<RMSE> (meV/atom) and force <MAE>/<RMSE> (meV/Å) averaged over 10 or five independent runs. Unless otherwise stated, values are test-set metrics from validation-selected models. The LODO results are also reported as mean and standard deviation across element folds.

### 3.1. Construction of Fine-Tuned Models with Randomly Partitioned Datasets

The randomly partitioned dataset, following an independent and identically distributed (IID) split, is used to assess model fitting accuracy and numerical stability under matched training and test distributions. In this setting, both training and test samples are drawn randomly from the same underlying data pool, thereby sharing the same statistical distribution. Model performance on the test sets is summarized in Table 1 for three training paradigms—the zero-shot, scratch-trained, and fine-tuned models—evaluated across small, medium, and large parameter scales on the equilibrium structure dataset (Eq, 420 samples). Additional results are provided for the fine-tuned model trained on the non-equilibrium structure dataset (NonEq, 6970 samples). Furthermore, we employ a trajectory-level group-split of the non-equilibrium dataset to mitigate the risk of information leakage. Specifically, all structures belonging to the same relaxation trajectory were assigned to the same subset. The results are very similar to those from the original random IID split. The small, medium, and large scales correspond to the officially released MACE-MP-0 model variants with trainable parameter counts of 3,847,696, 4,688,656, and 5,725,072, respectively.

On the equilibrium (Eq) structure dataset (Table 1), the fine-tuned model consistently attains substantially lower energy prediction errors across all model parameter scales, with <MAE> ranging from 7.34 to 17.07 meV/atom and <RMSE> from 13.20 to 30.31 meV/atom. In contrast, the model trained from scratch exhibits notably higher errors (<MAE>: 75.04–121.53 meV/atom; <RMSE>: 124.60–153.38 meV/atom), while the zero-shot model yields the largest deviations (<MAE>: 143.93–184.43 meV/atom; <RMSE>: 151.50–200.03 meV/atom). The energy prediction error of the fine-tuned model decreases monotonically as the model parameter size increases. In contrast, neither the scratch-trained model nor the zero-shot model exhibits a consistent scaling trend; this deviation is likely attributable to the limited size of the equilibrium dataset, which may hinder stable convergence and generalization across different model scales.

The fine-tuned model outperforms both the scratch-trained and zero-shot models at every scale examined in energy prediction. Specifically, the scratch-trained model shows energy <MAE> values that are 7.1–10.2 times higher, and <RMSE> values 5.1–10.0 times higher, than those of the fine-tuned model. Similarly, the zero-shot model produces <MAE> and <RMSE> values that are 10.8–19.6 times and 6.6–12.2 times greater, respectively, compared to the fine-tuned model. In addition to the mean errors, the standard deviations reported in Table 1 provide a measure of variability across independent runs. Overall, the fine-tuned models show relatively small standard deviations for both energy and force metrics, indicating that the observed performance trends are stable across different folds.

On the equilibrium (Eq) structure dataset (Table 1), the fine-tuned model achieves the lowest force prediction errors across all model parameter scales, with <MAE> ranging from 11.00 to 12.98 meV/Å and <RMSE> from 22.25 to 26.32 meV/Å. In comparison, the model trained from scratch exhibits generally higher and more variable errors (<MAE>: 3.57–17.08 meV/Å; <RMSE>: 5.91–31.17 meV/Å), while the zero-shot model yields the largest deviations (<MAE>: 44.55–69.44 meV/Å; <RMSE>: 88.57–146.13 meV/Å). Notably, although the scratch-trained model attains lower force errors in certain cases, it suffers from substantially higher energy errors compared to the fine-tuned model. This discrepancy is likely attributable to convergence issues arising from training on limited equilibrium data across varying model sizes. Across training paradigms, the fine-tuning strategy generally provides the best energy–force balance.

The fine-tuned model delivers generally more consistent and accurate force predictions than both the scratch-trained and zero-shot approaches. Relative to the fine-tuned model, the scratch-trained model shows force <MAE> values that range from 0.3 times lower to 1.3 times higher, and <RMSE> values that are 0.3 times lower to 1.2 times higher. In contrast, the zero-shot model exhibits markedly larger errors, with force <MAE> and <RMSE> values approximately 4.1–5.3 times and 4.0–5.6 times greater, respectively.

Incorporating extensive non-equilibrium structural data (NonEq) substantially enhances the prediction accuracy of the fine-tuned model across all parameter scales. The fine-tuned model trained with non-equilibrium data (FT-E_NE) achieves energy prediction errors in the range of 2.27–5.55 meV/atom (<MAE>) and 3.79–7.47 meV/atom (<RMSE>), and force prediction errors of 13.83–18.28 meV/Å (<MAE>) and 24.26–31.58 meV/Å (<RMSE>). In comparison, the model fine-tuned solely on equilibrium data (FT-E) exhibits notably larger energy errors. Compared to the FT-E_NE model, the FT-E model shows energy <MAE> and <RMSE> values that are approximately 3.1–3.2 times and 3.5–4.1 times higher, respectively. To further examine whether the observed difference is solely caused by dataset size, we performed a size-matched ablation by randomly sampling subsets from the non-equilibrium dataset to match the number of equilibrium structures. The comparison results show that the enlarged dataset size by including non-equilibrium structures indeed benefits the prediction accuracy. At the same matched dataset scale, fine-tuning with non-equilibrium data still yields slightly better energy prediction, and the improvement becomes clearer as the dataset scale increases. It should be noted that non-equilibrium data are inevitably generated during geometry relaxation when obtaining equilibrium structures. Therefore, including these existing non-equilibrium data in the training set adds little extra cost, while helping improve the accuracy of ML models. From a practical point of view, using both equilibrium and non-equilibrium data is a better strategy for achieving more accurate predictions.

However, for force prediction, the FT-E model achieves lower errors than the FT-E_NE model, with <MAE> and <RMSE> values ~0.7–0.8 and 0.8–0.9 times those of the FT-E_NE model, respectively. Across all model parameter scales, force prediction accuracy on the equilibrium structure dataset (Eq) consistently surpasses that on the non-equilibrium dataset (Non-Eq). Specifically, the force <MAE> values for FT-E are 11.00, 11.11, and 12.98 meV/Å at small, medium, and large scales, while the corresponding values for FT-E_NE are 18.28, 15.69, and 13.83 meV/Å. Beyond differences in dataset size, this performance gap likely stems from distinct force distribution characteristics. This difference is mainly associated with the distinct force distributions of the two datasets. In the FT-E dataset, most structures are equilibrium or near-equilibrium configurations, so the atomic forces are generally small and near zero. In contrast, the FT-E_NE dataset contains a broader range of non-equilibrium structures with more widely distributed and frequently non-zero force values. Therefore, force prediction on FT-E_NE is intrinsically more challenging and can naturally result in somewhat larger absolute force errors, even though the model exhibits better energy performance.

**Figure 3:**
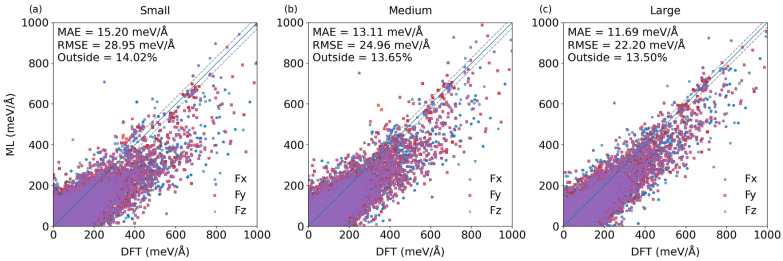
Parity plots of atomic forces in the non-equilibrium structure dataset (Non-Eq) predicted by the fine-tuned MACE model (FT-E_NE) versus DFT references for three model sizes: (**a**) small, (**b**) medium, and (**c**) large parameter scales. The dashed lines indicate deviations exceeding 100% of the MAE.

Figure 3 displays parity plots comparing predicted force components in the non-equilibrium structure dataset from the representative fine-tuned models (FT-E_NE) against reference DFT calculations, evaluated across three model parameter scales: small, medium, and large. Each plot shows individual data points for the three Cartesian force components (F_x_, F_y_, F_z_), represented by distinct colors, with a diagonal line indicating perfect agreement. The three force components show comparable levels of accuracy across all scales, with no consistent bias observed toward any particular direction. Across all scales, the model demonstrates strong predictive capability, with the vast majority of data points clustering tightly along the parity line. The overall error metrics (MAE and RMSE) remain relatively stable and decrease with parameter scales, ranging from 11.69 to 15.20 meV/Å for MAE and 22.20–28.95 meV/Å for RMSE.

The machine learning model exhibits consistent and high-quality force prediction across varying model scales, with the better performance observed at the larger scale as discussed below.

(a) Small-scale model: The prediction errors are higher than in the other scales, with MAE at 15.20 meV/Å and RMSE at 28.95 meV/Å. While the scatter is still reasonably contained, a few outliers appear at higher force magnitudes. Here, the outliers (65,357 outlier points among 466,056 in total) are defined as data points whose absolute force error exceeds 100% of the corresponding MAE value.

(b) Medium-scale model: This medium-scale model yields a modest overall error, with MAE = 13.11 meV/Å and RMSE = 24.96 meV/Å. The data points are more densely clustered around the parity line, indicating improved model precision compared to the small-scale model.

(c) Large-scale model: The large-scale model leads to the lowest overall error, with MAE = 11.69 meV/Å and RMSE = 22.20 meV/Å. The scatter of points is denser and closer to the parity line, especially at higher force magnitudes (>600 meV/Å), suggesting the large parameter scale improves prediction accuracy for extreme force values.

Overall, the combination of large-scale model parameters and non-equilibrium training data yields the best performance, achieved by the FT-E_NE (large) model. This optimal model attains the lowest errors in this study, with energy prediction <MAE> and <RMSE> of 2.27 meV/atom and 3.79 meV/atom, respectively, and force prediction <MAE> and <RMSE> of 13.83 meV/Å and 24.26 meV/Å, respectively.

**Figure 4:**
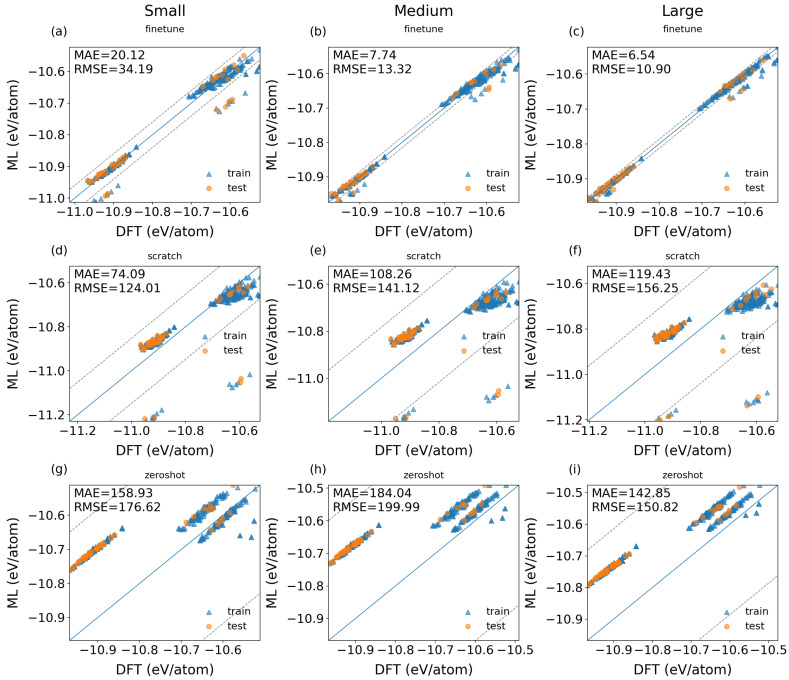
Energy predicted by ML vs. DFT references for the randomly partitioned test dataset (IID) of equilibrium structures (Eq) using three ML models at different parameter scales: (**a**–**c**) the fine-tuned models at small, medium, and large parameter scales, respectively; (**d**–**f**) the scratch models at small, medium, and large parameter scales, respectively; (**g**–**i**) the zero-shot inference models at small, medium, and large parameter scales, respectively. Each panel is a representative fold whose errors are close to the mean values by comparing predicted and reference energies. The dashed lines indicate deviations exceeding 100% of the MAE.

Figure 4 compares the energy predictions of three machine learning models at varying parameter scales against DFT reference values for the randomly partitioned test dataset (IID) of equilibrium structures. For each model configuration, the displayed panel corresponds to the fold whose evaluation metrics align most closely with the mean values reported in Table 1. The fine-tuned models at all three scales demonstrate substantially closer agreement with the reference data, as evidenced by their tighter clustering around the y = x line. In contrast, both the scratch-trained and zero-shot models exhibit more pronounced systematic offsets and heavier distribution tails.

As shown in Figure 4a–c, the fine-tuned model provides reasonably accurate predictions even at the smallest scale, with only slight systematic deviations. The outliers in Figure 4 are defined as data points whose absolute energy error exceeds 100% of the corresponding MAE value; the outlier fractions for the fine-tuned small, medium, and large-scale models are 14.3%, 9.6%, and 12.0%, respectively. In comparison, the scratch-trained model [Figure 4d–f] captures the general trend for most data points but produces a notable number of random high-error outliers. These outliers persist across all parameter scales and are not eliminated by increasing model capacity. The zero-shot model [Figure 4g–i] displays the most severe systematic bias, which remains largely unchanged even when model size is increased.

### 3.2. Verification of Fine-Tuned Models with Leave-One-Dopant-Out Partitioned Datasets

The leave-one-dopant-out (LODO) data partition protocol evaluates transfer robustness across held-out dopant elements. Table 2 presents the LODO test results for fine-tuned MACE models (FT-E) at a medium scale on a series of Mo-based alloys, evaluated under two data-splitting protocols: strict and relaxed. A clear performance gap is observed between the two protocols. Under the strict protocol, the mean energy MAE is substantially higher (76.98 meV/atom) and exhibits large variability (±36.05 meV/atom), indicating poor generalization to completely unseen doped alloying elements. The force predictions are also less accurate, with a mean MAE of 41.0 meV/Å. In stark contrast, the relaxed protocol yields a drastic improvement: the mean energy MAE drops to 20.03 meV/atom, and force MAE is more than halved to 18.8 meV/Å. Variability in errors is also significantly reduced. However, this setting should be interpreted as a partial-exposure transfer test rather than a strict unseen-element OOD benchmark.

The Relaxed-LODO mean statistics are strongly affected by the outlier Cr (energy MAE = 136.15 meV/atom). Excluding the Cr element, we recomputed protocol means. Under the Strict-LODO protocol the averaged energy RMSE over the remaining elements is 80.23 meV/atom, whereas the Relaxed-LODO mean is 10.69 meV/atom (Table 2). This corresponds to an 86.67% reduction from Strict-LODO to Relaxed-LODO.

Notably, the performance disparity is consistent through most elements except for a few element-dependent cases. For most elements (e.g., Fe, Mn, Nb, Re, Ta, Ti, V, W), the relaxed protocol leads to a massive reduction in energy errors—often by an order of magnitude. Their energy MAE under the strict protocol ranges from ~54 to ~132 meV/atom, but falls to below ~10 meV/atom under the relaxed protocol. This result indicates that allowing partial exposure of the held-out dopant in double-site environments can significantly improve transfer performance, but it should not be interpreted as evidence of genuine unseen-element extrapolation.

In contrast, for Cr and Y, the relaxed protocol results in comparable or even slightly worse performance compared to the strict protocol, suggesting these elements may possess distinct chemical or structural features that are not well-represented in the training data of the other splits. Based on the LODO test results in Table 2, the elements Cr and Y exhibit a markedly different performance trend compared to the other dopants (Fe, Mn, Nb, Re, Ta, Ti, V, W) when moving from the strict to the Relaxed-LODO protocol.

For Chromium (Cr), its energy MAE increases from 84.01 meV/atom (strict) to 136.15 meV/atom (relaxed), and force MAE also rises significantly (Table 2). This indicates the model’s performance degrades for Cr when trained on data that includes other, dissimilar dopants. This suggests that Cr is a particularly challenging case for the present model. A possible reason is that Cr may involve more complex local electronic or magnetic effects, which are harder for the current MLIP framework to capture [49,50]. However, since no direct magnetic analysis was performed in this work, this explanation should be regarded as a plausible hypothesis rather than a proven mechanism.

For Yttrium (Y), while it shows the lowest error under the strict protocol (6.80 meV/atom), its error under the relaxed protocol increases to 10.03 meV/atom (Table 2). Unlike the others, Y’s error does not improve with the more permissive training data; it gets slightly worse. Y is chemically distinct as a larger and more electropositive rare-earth element. Its size, charge, and bonding nature (with possible stronger ionic character) are fundamentally different from the smaller transition metal dopants. While the model can learn a specific mapping for Y alone (strict protocol), incorporating the interaction with the other elements (relaxed protocol) introduces a conflicting signal that degrades the model’s specific performance for Y, though not as catastrophically as for Cr.

In summary, the fine-tuned MACE model demonstrates strong and stable predictive capability under the Relaxed-LODO protocol whose training data is chemically informative and generalizable successfully to most unseen alloying elements with high accuracy. The strict protocol, however, reveals a significant generalization challenge, highlighting a strong model dependence on the specific dopant seen during training. The exceptional cases (e.g., Cr and Y) under the relaxed protocol appear to be non-transferable as outliers in the chemical/feature space defined by the other dopants in this alloy system, which warrant further investigation into their unique bonding environments or potential data representation gaps.

**Figure 5:**
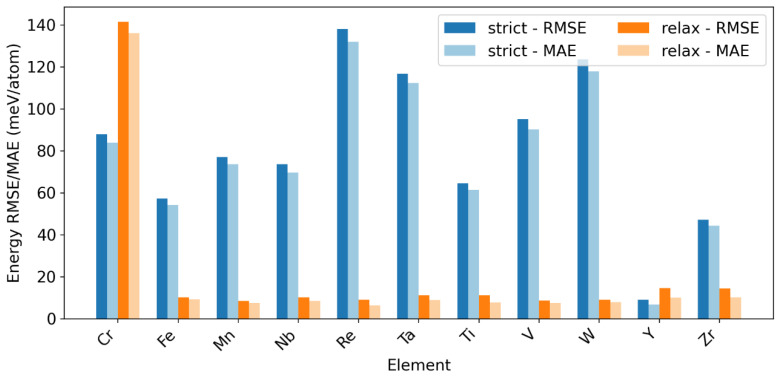
Energy errors (MAE and RMSE in meV/atom) predicted by the fine-tuned MACE models (FT-E) at a medium scale for various dopant elements in Mo-based alloys under two leave-one-dopant-out (LODO) partitioning protocols: “relax” (blue) and “strict” (orange).

Figure 5 illustrates the energy prediction error (MAE and RMSE) using the fine-tuned MACE models for various dopant elements in Mo-based alloys under two leave-one-dopant-out (LODO) partitioning protocols: Strict-LODO and Relaxed-LODO.

For the majority of dopant elements—including Fe, Mn, Nb, Re, Ta, Ti, V, and W—the model exhibits markedly improved predictive accuracy under the Relaxed-LODO protocol compared to the Strict-LODO protocol. This difference reflects the benefit of partial dopant exposure in the relaxed setting. Therefore, the Relaxed-LODO results are better interpreted as transfer under partially shared chemical environments, rather than as a strict benchmark of unseen-element extrapolation.

Quantitatively, under the strict protocol, energy RMSE values for these elements range from ~57 meV/atom (Fe) to 138 meV/atom (Re). In contrast, under the relaxed protocol, the same elements show RMSE values between 6.46 meV/atom (Re) and 18.28 meV/atom (W), indicating a reduction by roughly one order of magnitude on average. This dramatic improvement underscores the effectiveness of the relaxed protocol in stabilizing predictions for well-behaved dopant systems.

Overall, the chart in Figure 5 reveals a consistent trend across most dopant elements, with notable exceptions that warrant separate discussion below except for Cr and Y discussed earlier.

(i) Niobium (Nb): Exhibits moderate RMSE under the strict protocol (~73.65 meV/atom) but achieves one of the lowest RMSE under the relaxed protocol (~8.56 meV/atom). Its performance improvement is among the most pronounced, suggesting that Nb benefits significantly from the increased data heterogeneity introduced by the relaxed partitioning.

(ii) Rhenium (Re): Despite having the highest RMSE under the strict protocol (~138.02 meV/atom), Re shows the lowest RMSE under the relaxed protocol (~6.46 meV/atom). This exceptional improvement implies that Re’s predictive difficulty under strict conditions stems primarily from limited or biased training representation, which is effectively alleviated in the relaxed setting.

(iii) Tantalum (Ta): Demonstrates a relatively high strict-error (~116.76 meV/atom) but drops sharply to ~8.89 meV/atom under relaxation. The magnitude of improvement is comparable to Re and Nb, indicating that Ta’s model performance is highly sensitive to the data partitioning strategy.

(iv) Tungsten (W): Although its strict-error (~123.69 meV/atom) is among the highest, its relaxed-error (~8.01 meV/atom) is among the lowest. W’s behavior mirrors that of Re and Ta, reinforcing the pattern that heavy refractory metals (Re, Ta, W) exhibit high sensitivity to data partitioning, with large gains achievable under relaxed conditions.

**Figure 6:**
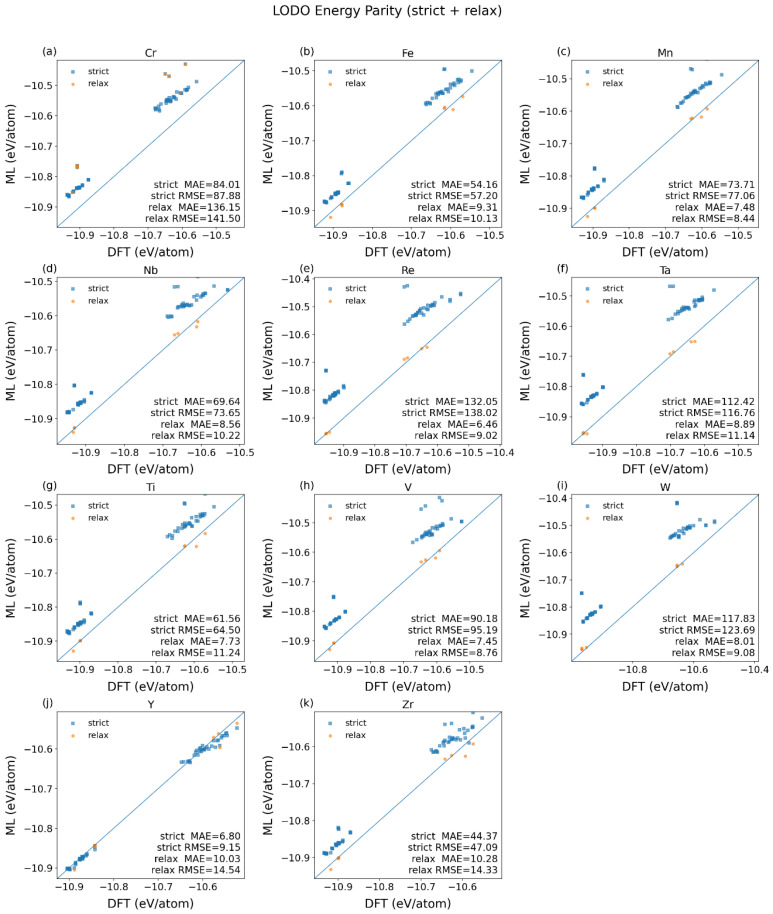
Energy parity plots for each new held-out element in the equilibrium structure dataset (Eq) predicted by the fine-tuned MACE model (FT-E) at a medium scale versus DFT references under Strict-LODO and Relaxed-LODO data protocols. (**a**) Cr; (**b**) Fe; (**c**) Mn; (**d**) Nb; (**e**) Re; (**f**) Ta; (**g**) Ti; (**h**) V; (**i**) W; (**j**) Y; (**k**) Zr.

The discussion above can be further supported by the energy parity plots in Figure 6 for each held-out element in the equilibrium structure dataset (Eq) predicted by the fine-tuned MACE model (FT-E) at a medium scale compared with the DFT references under Strict-LODO and Relaxed-LODO data protocols. It shows that the inclusion of Cr in the relaxed training dataset did not help to improve the prediction performance [Figure 6a], while the element Y behaves well in both the strict and relaxed cases [Figure 6j]. The other alloying elements show systematic deviation from the diagonal lines in the strict cases but recover back to the references in the relaxed cases, which indicates the partially seen elements in the training period are critical to the good prediction performance.

In summary, excluding the anomalous behavior of Cr and Y, the remaining dopant elements demonstrate a clear and favorable response to the Relaxed-LODO protocol. Most notably, Re, Ta, and W—despite exhibiting high errors under strict partitioning—achieve state-of-the-art prediction accuracy under relaxation, highlighting the importance of data diversity for modeling these elements. Nb also shows substantial improvement, confirming that the relaxed protocol enhances generalization across a broad range of dopant chemistries. This consistent trend supports the conclusion that relaxed partitioning is a critical strategy for achieving robust and accurate energy predictions in Mo-based alloy systems, particularly for elements that are otherwise poorly represented or inherently complex under strict training conditions.

Overall, both the random partition (IID) and element-wise partition (LODO) results show that the fine-tuned MACE provides practical accuracy for both in-distribution fitting and cross-element transfer. Under IID, absolute errors reach the meV/atom level (energy MAE: 2.27–17.07 meV/atom). Under LODO, after excluding the Cr element, the mean energy MAE is 76.27 meV/atom for Strict-LODO and 8.42 meV/atom for Relaxed-LODO. Based on these results, the following application study uses the fine-tuned MACE as the primary model and examines reliability boundaries for new elements against DFT references.

### 3.3. Transferability of Fine-Tuned Models to Unknown Alloying Elements

To assess the transferability of the fine-tuned models to unknown alloying elements in novel alloy design, this work evaluates nine substitutional elements not present in the original training set: Al, Zn, Cu, Ag, Au, Hg, Co, Ni, and Hf. A total of 79 non-equilibrium structures were generated from single-site substitution systems in a BCC Mo matrix via geometric relaxation. These elements were chosen to extend coverage to a broad range of transition metals with d-orbital electrons, complementing the original training set of 11 alloying elements, and to include the industrially relevant p-block element Al. Model predictions are benchmarked against DFT reference calculations.

First, we examine the capability of the previous fine-tuned MACE model (FT-E) at a medium scale to directly predict the unknown doped alloys without changing their architecture and parameters. The fine-tuned MACE model contains embeddings only for the element types observed during the training stage of the fine-tuning process and does not provide valid representations for the unseen atomic numbers. Therefore, the unseen elements are mapped to known elements with close atomic numbers, period, or group in the periodic table and their corresponding embeddings are reused as a proxy setting. Concretely, each unseen element is initialized by a proxy embedding from a training-set element. This proxy step is used only for embedding initialization at inference and does not alter the test-set settings. This prediction strategy is essentially a direct inference with proxy initialization via mapping of the known elements, denoted as DIPI. This DIPI approach probes engineering-level applicability under elemental extrapolation but does not constitute strict chemical-identity modeling of truly unseen elements.

**Figure 7:**
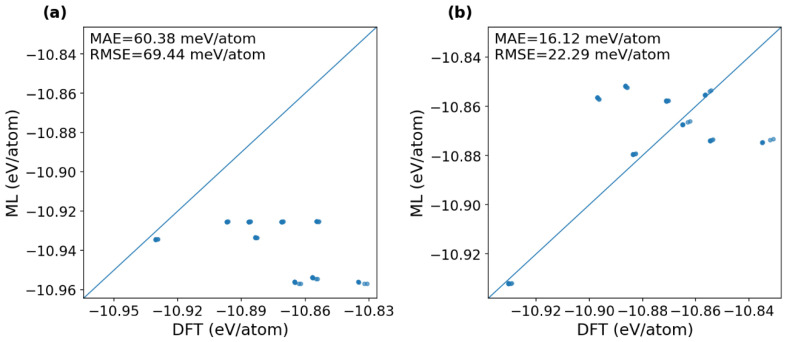
Energy parity comparison for two new-element evaluation strategies. (**a**) DIPI (direct inference with proxy initialization) with MAE = 60.38 meV/atom, RMSE = 69.44 meV/atom; (**b**) refine-tuned model with MAE = 16.12 meV/atom, RMSE = 22.29 meV/atom.

Figure 7a presents the prediction results obtained via the direct-inference (DIPI) approach. The data points predominantly lie below the y = x line, forming a distinct banded pattern that reflects a systematic underestimation of the new-element samples. The corresponding energy errors remain substantial, with an MAE of 60.38 meV/atom and an RMSE of 69.44 meV/atom. Nevertheless, these errors are notably lower than those of the scratch-trained model (MAE = ~112 meV/atom, RMSE = ~144 meV/atom) and the zero-shot model (MAE = ~184 meV/atom, RMSE = ~200 meV/atom). The observed degradation in accuracy arises primarily from element-identity aliasing induced by the proxy-mapping procedure. Since the model cannot explicitly distinguish the electronic-structure differences associated with actual atomic numbers, it provides only approximate, surrogate responses in chemically similar environments. Consequently, the direct-inference results should be interpreted as a conservative estimate of the current model’s capability rather than a rigorous upper bound for predictions on unseen elements.

**Figure 8:**
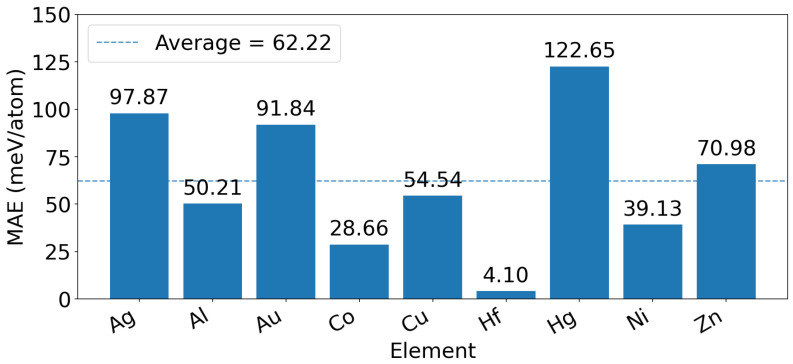
Histogram of element-wise MAE values for the DIPI predictions on the nine unseen alloying elements.

To further clarify the transferability of the DIPI strategy across different unseen elements, we added the element-wise MAE distribution in Figure 8. The results show a pronounced element dependence in the direct proxy-based inference errors. Among the nine unseen alloying elements, Hf exhibits the lowest error (4.10 meV/atom), while Co and Ni also remain at relatively low levels (28.66 and 39.13 meV/atom, respectively). By contrast, Hg shows the largest error (122.65 meV/atom), and Ag and Au also display high errors (97.87 and 91.84 meV/atom, respectively). The remaining elements, including Al, Cu, and Zn, fall in an intermediate range of approximately 50–71 meV/atom. These results indicate that the performance of DIPI strongly depends on how well the proxy embedding can approximate the true chemical identity of the unseen dopant.

Figure 7b shows the predictions of the updated model after refinement with limited DFT data for the nine newly introduced alloying elements under an IID train/validation/test split. This setting therefore represents rapid adaptation with limited new-element data. The scatter points lie close to the y = x line, with an MAE of 16.12 meV/atom and an RMSE of 22.29 meV/atom. Although these errors are higher than those of the original fine-tuned model on the equilibrium set (FT-Eq, medium scale: MAE = 8.10 meV/atom, RMSE = 12.45 meV/atom), they represent a 73.3% reduction in MAE and a 67.9% reduction in RMSE compared to the direct-inference approach. This comparison demonstrates that refine-tuning substantially improves reliability for unseen-element prediction, mitigating the strong element-identity aliasing errors inherent in direct proxy-based inference.

Potential improvements for enhancing transferability to unseen elements include: explicitly incorporating new elements during pretraining or fine-tuning, expanding the set of learnable element embeddings, and performing rapid recalibration with a small amount of additional DFT data for the new elements. Here, we adopt an independent and identically distributed (IID) data partition protocol, randomly splitting all samples into training, validation, and test sets in an (81:9):10 ratio. This IID split ensures that all new elements appear in the training data, while the test set contains only the new elements, ~50 structures across the nine alloying elements. The random partitioning was repeated independently 10 times, and the previously fine-tuned models were retrained on each split; these updated models are denoted as refine-tuned models. The reported errors are averaged over the 10 independent runs.

## 4. Conclusions

Machine learning interatomic potentials (MLIPs) are typically developed for globally homogeneous crystal systems that exhibit only minor local distortions around equilibrium positions. In contrast, multicomponent engineering alloys often contain substitutional alloying elements that are distributed non-uniformly within the matrix, resulting in locally heterogeneous chemical short-range order. To address this, we developed a fine-tuned MLIP based on the MACE foundation model, specifically designed for dilute Mo-based alloys doped with 20 substitutional elements: Cr, Fe, Mn, Nb, Re, Ta, Ti, V, W, Y, Zr, Al, Zn, Cu, Ag, Au, Hg, Co, Ni, and Hf. The MLIP model was trained on over 7000 equilibrium and non-equilibrium structures obtained from first-principles density functional theory (DFT) calculations.

The optimized large-scale fine-tuned model achieves state-of-the-art accuracy, with mean absolute errors (MAE) of 2.27 meV/atom for energy and 13.83 meV/Å for force prediction, and corresponding root-mean-square errors (RMSE) of 3.79 meV/atom and 24.26 meV/Å, respectively. We systematically evaluated the transferability of the fine-tuned models using different dopant-level partition protocols. The results show that unknown-element extrapolation remains challenging under the stricter Strict-LODO setting, whereas substantially improved performance can be achieved under the less restrictive Relaxed-LODO partial-exposure transfer setting. Validation tests strongly recommend incorporating a limited amount of new-element data to refine the model, which reduces the energy MAE to below approximately 20 meV/atom.

The fine-tuned models outperform scratch-trained models (trained without pretrained weights) by a factor of 7–10 in MAE, and surpass zero-shot foundation models (without fine-tuning) by a factor of 10–20. This substantial improvement is consistently observed across different dataset sizes (equilibrium vs. non-equilibrium structures) and model parameter scales. Our work demonstrates the necessity and effectiveness of transfer learning from globally representative systems to locally ordered alloy environments, offering a practical MLIP tool for the intelligent design of multicomponent alloys. However, direct transfer to elements with unusual magnetic or electronic behavior remains challenging. Future improvement may rely on foundation models with explicit magnetic treatment [51,52].

## Figures and Tables

**Table 1:** Performance metrics, averaged <MAE> and <RMSE> of energies and forces with standard deviation, across the various training paradigms and model scales under the randomly partitioned test dataset (IID).

ML Models	Energy <MAE> (meV/Atom)	Energy <RMSE> (meV/Atom)	Force <MAE> (meV/Å)	Force <RMSE> (meV/Å)
FT-E(S)	17.1 ± 4.7	30.3 ± 11.4	11.0 ± 0.8	22.8 ± 1.7
FT-E(M)	8.1 ± 1.4	12.5 ± 2.8	11.1 ± 0.7	22.3 ± 1.7
FT-E(L)	7.3 ± 1.7	13.2 ± 4.0	13.0 ± 1.2	26.3 ± 2.6
FT-E_NE(S)	5.6 ± 0.1	7.5 ± 0.5	18.3 ± 0.3	31.6 ± 0.8
FT-E_NE(M)	3.5 ± 0.1	5.0 ± 0.8	15.7 ± 0.2	27.3 ± 0.6
FT-E_NE(L)	2.3 ± 0.1	3.8 ± 0.9	13.8 ± 0.2	24.3 ± 0.5
FT-E_NE (S_group_split)	6.0 ± 0.9	8.1 ± 0.9	18.9 ± 1.4	32.9 ± 2.3
FT-E_NE (M_group_split)	3.7 ± 1.3	5.4 ± 1.9	16.1 ± 1.4	28.4 ± 2.3
FT-E_NE (L_group_split)	1.9 ± 0.1	3.4 ± 1.0	13.3 ± 0.1	24.0 ± 0.5
FT-E_NE (S_size-match)	12.3 ± 2.1	16.1 ± 3.0	24.8 ± 2.2	45.6 ± 8.7
FT-E_NE (M_size-match)	7.9 ± 1.3	10.4 ± 1.7	22.7 ± 1.8	43.0 ± 8.6
FT-E_NE (L_size-match)	5.0 ± 1.3	7.7 ± 2.4	21.6 ± 1.8	40.8 ± 6.3
Scratch-E(S)	75.0 ± 22.0	124.6 ± 28.0	17.1 ± 3.0	31.2 ± 5.6
Scratch-E(M)	112.0 ± 11.9	144.4 ± 20.9	11.3 ± 2.5	21.0 ± 4.6
Scratch-E(L)	121.5 ± 8.6	153.4 ± 18.8	3.6 ± 1.1	5.9 ± 1.9
Zero-shot-E(S)	159.2 ± 6.9	176.5 ± 4.6	69.4 ± 5.5	146.1 ± 7.9
Zero-shot-E(M)	184.4 ± 7.0	200.0 ± 5.0	50.3 ± 3.5	100.6 ± 4.8
Zero-shot-E(L)	143.9 ± 4.4	151.5 ± 3.4	44.6 ± 3.3	88.6 ± 4.6

**Table 2:** MAE (meV/atom) and RMSE (meV/Å) results of the leave-one-dopant-out (LODO) test dataset with strict and relaxed partition protocols for each alloying element in Mo-based alloys predicted by the fine-tuned MACE models (FT-E) at a medium scale. MeanAll represents the averaged values of energy and force MAE or RMSE across all dopant elements, while MeanNC represents the corresponding averaged values excluding the outlier Cr.

Element	Strict-LODO				Relaxed-LODO			
	Energy MAE	Energy RMSE	Force MAE	Force RMSE	Energy MAE	Energy RMSE	Force MAE	Force RMSE
Fe	54.16	57.20	50.3	108.8	9.31	10.13	17.8	41.9
Mn	73.71	77.06	45.5	93.4	7.48	8.44	15.3	29.6
Nb	69.64	73.65	36.2	74.4	8.56	10.22	9.0	14.8
Re	132.05	138.02	40.9	69.3	6.46	9.02	12.1	23.8
Ta	112.42	116.76	28.0	60.8	8.89	11.14	11.0	19.1
Ti	61.56	64.50	31.4	69.6	7.73	11.24	10.2	19.3
V	90.18	95.19	34.6	71.8	7.45	8.76	11.8	23.6
W	117.84	123.69	49.4	118.9	8.01	9.09	9.9	18.8
Y	6.80	9.15	43.7	74.8	10.03	14.54	18.5	32.4
Zr	44.37	47.09	37.1	71.2	10.28	14.33	13.9	24.3
Cr	84.01	87.88	54.1	126.8	136.15	141.50	77.4	177.2
MeanNC	76.27	80.23	39.71	81.30	8.42	10.69	12.95	24.76
MeanAll Std.	76.98 ± 36.05	80.93 ± 37.18	41.0 ± 8.3	85.4 ± 22.8	20.03 ± 38.53	22.58 ± 39.49	18.8 ± 19.7	38.6 ± 46.6

## Data Availability

The data presented in this study are available on request from the corresponding author. (the data are not publicly available due to privacy).

## Supplementary Materials

The following supporting information can be downloaded at: https://www.mdpi.com/article/10.3390/ma19091715/s1, Text S1: Architecture of MACE models. Figure S1: Schematic illustration of the MACE architecture. The pipeline follows Structure → Atomic Environment → embedding → (Interaction–Product–Update, repeated for *K* layers) → learnable atomic features $f_{i}$→ Readout → atomic energy $E_{i}$. Text S2: Computational parameters in the first-principles calculations. Table S1: Overview of the ML models and their settings including training paradigms, data splitting, losses, and metrics in this work. References [11,17,47,48,53,54,55,56,57,58,59,60,61] are cited in the Supplementary Materials.

## References

[1] Xu H., Cui T., Tang C., Ma J., Zhou D., Li Y., Gao X., Gong X., Ouyang W., Zhang S. (2025). Evidential Deep Learning for Interatomic Potentials. Nat. Commun..

[2] Yang Z., Wang X., Li Y., Lv Q., Chen C.Y.-C., Shen L. (2025). Efficient Equivariant Model for Machine Learning Interatomic Potentials. npj Comput. Mater..

[3] Ran N., Yin L., Qiu W., Liu J. (2024). Recent Advances in Machine Learning Interatomic Potentials for Cross-Scale Computational Simulation of Materials. Sci. China Mater..

[4] Su T., Hu S., Wu Y., Oyang R., Wang X., Li M., Reimers J., Zhang T.-Y. (2026). DeePAW: A Universal Machine Learning Model for Orbital-Free Ab Initio Calculations. arXiv.

[5] Chen Z., Yang D., Li X., Li J., Yuan H., Cui J. (2025). Machine-Learning-Assisted Multi-Element Optimization of Mechanical Properties in Spinel Refractory Materials. Materials.

[6] Behler J., Parrinello M. (2007). Generalized Neural-Network Representation of High-Dimensional Potential-Energy Surfaces. Phys. Rev. Lett..

[7] Bartók A.P., Payne M.C., Kondor R., Csányi G. (2010). Gaussian Approximation Potentials: The Accuracy of Quantum Mechanics, without the Electrons. Phys. Rev. Lett..

[8] Bartók A.P., Kondor R., Csányi G. (2013). On Representing Chemical Environments. Phys. Rev. B.

[9] Thompson A.P., Swiler L.P., Trott C.R., Foiles S.M., Tucker G.J. (2015). Spectral Neighbor Analysis Method for Automated Generation of Quantum-Accurate Interatomic Potentials. J. Comput. Phys..

[10] Shapeev A.V. (2016). Moment Tensor Potentials: A Class of Systematically Improvable Interatomic Potentials. Multiscale Model. Simul..

[11] Drautz R. (2019). Atomic Cluster Expansion for Accurate and Transferable Interatomic Potentials. Phys. Rev. B.

[12] Schütt K., Kindermans P.-J., Sauceda Felix H.E., Chmiela S., Tkatchenko A., Müller K.-R. (2017). SchNet: A Continuous-Filter Convolutional Neural Network for Modeling Quantum Interactions. Advances in Neural Information Processing Systems.

[13] Gasteiger J., Groß J., Günnemann S. (2022). Directional Message Passing for Molecular Graphs. arXiv.

[14] Gasteiger J., Becker F., Günnemann S. (2021). Gemnet: Universal Directional Graph Neural Networks for Molecules. Adv. Neural Inf. Process. Syst..

[15] Schütt K., Unke O., Gastegger M. Equivariant Message Passing for the Prediction of Tensorial Properties and Molecular Spectra. Proceedings of the International Conference on Machine Learning.

[16] Batzner S., Musaelian A., Sun L., Geiger M., Mailoa J.P., Kornbluth M., Molinari N., Smidt T.E., Kozinsky B. (2022). E(3)-Equivariant Graph Neural Networks for Data-Efficient and Accurate Interatomic Potentials. Nat. Commun..

[17] Batatia I., Kovacs D.P., Simm G., Ortner C., Csányi G. (2022). MACE: Higher Order Equivariant Message Passing Neural Networks for Fast and Accurate Force Fields. Adv. Neural Inf. Process. Syst..

[18] Gasteiger J., Shuaibi M., Sriram A., Günnemann S., Ulissi Z., Zitnick C.L., Das A. (2022). GemNet-OC: Developing Graph Neural Networks for Large and Diverse Molecular Simulation Datasets. arXiv.

[19] Chen C., Ong S.P. (2022). A Universal Graph Deep Learning Interatomic Potential for the Periodic Table. Nat. Comput. Sci..

[20] Deng B., Zhong P., Jun K., Riebesell J., Han K., Bartel C.J., Ceder G. (2023). CHGNet as a Pretrained Universal Neural Network Potential for Charge-Informed Atomistic Modelling. Nat. Mach. Intell..

[21] Batatia I., Benner P., Chiang Y., Elena A.M., Kovács D.P., Riebesell J., Advincula X.R., Asta M., Avaylon M., Baldwin W.J. (2025). A Foundation Model for Atomistic Materials Chemistry. J. Chem. Phys..

[22] Yang H., Hu C., Zhou Y., Liu X., Shi Y., Li J., Li G., Chen Z., Chen S., Zeni C. (2024). MatterSim: A Deep Learning Atomistic Model Across Elements, Temperatures and Pressures. arXiv.

[23] Ma J., Fu X., Xie W., Hu P. (2026). From Pretrained to Precision: Fine-Tuning Universal Interatomic Potentials for Accurate Catalytic Reaction Simulations. J. Chem. Theory Comput..

[24] Zhang L., Han J., Wang H., Car R., E W. (2018). Deep Potential Molecular Dynamics: A Scalable Model with the Accuracy of Quantum Mechanics. Phys. Rev. Lett..

[25] Musaelian A., Batzner S., Johansson A., Sun L., Owen C.J., Kornbluth M., Kozinsky B. (2023). Learning Local Equivariant Representations for Large-Scale Atomistic Dynamics. Nat. Commun..

[26] Chanussot L., Das A., Goyal S., Lavril T., Shuaibi M., Riviere M., Tran K., Heras-Domingo J., Ho C., Hu W. (2021). Open Catalyst 2020 (OC20) Dataset and Community Challenges. ACS Catal..

[27] Tran R., Lan J., Shuaibi M., Wood B.M., Goyal S., Das A., Heras-Domingo J., Kolluru A., Rizvi A., Shoghi N. (2023). The Open Catalyst 2022 (OC22) Dataset and Challenges for Oxide Electrocatalysts. ACS Catal..

[28] Kirklin S., Saal J.E., Meredig B., Thompson A., Doak J.W., Aykol M., Rühl S., Wolverton C. (2015). The Open Quantum Materials Database (OQMD): Assessing the Accuracy of DFT Formation Energies. npj Comput. Mater..

[29] Radova M., Stark W.G., Allen C.S., Maurer R.J., Bartók A.P. (2025). Fine-Tuning Foundation Models of Materials Interatomic Potentials with Frozen Transfer Learning. npj Comput. Mater..

[30] Smith J.S., Nebgen B.T., Zubatyuk R., Lubbers N., Devereux C., Barros K., Tretiak S., Isayev O., Roitberg A.E. (2019). Approaching Coupled Cluster Accuracy with a General-Purpose Neural Network Potential through Transfer Learning. Nat. Commun..

[31] Wu Z., Zhou L., Hou P., Liu Y., Wang R., Guo T., Liu J.-C. (2025). A Machine Learning Interatomic Potential Data Set and Model for Catalysis with Local Fine-Tuning to Chemical Accuracy. JACS Au.

[32] Kulichenko M., Nebgen B., Lubbers N., Smith J.S., Barros K., Allen A.E.A., Habib A., Shinkle E., Fedik N., Li Y.W. (2024). Data Generation for Machine Learning Interatomic Potentials and Beyond. Chem. Rev..

[33] Wang G., Wang C., Zhang X., Li Z., Zhou J., Sun Z. (2024). Machine Learning Interatomic Potential: Bridge the Gap between Small-Scale Models and Realistic Device-Scale Simulations. iScience.

[34] Deng B., Choi Y., Zhong P., Riebesell J., Anand S., Li Z., Jun K., Persson K.A., Ceder G. (2025). Systematic Softening in Universal Machine Learning Interatomic Potentials. npj Comput. Mater..

[35] Liu Y., Mo Y. (2024). Learning from Models: High-Dimensional Analyses on the Performance of Machine Learning Interatomic Potentials. npj Comput. Mater..

[36] Li Y., Xiao B., Tang Y., Liu F., Wang X., Yan F., Liu Y. (2020). Center-Environment Feature Model for Machine Learning Study of Spinel Oxides Based on First-Principles Computations. J. Phys. Chem. C.

[37] Tang Y., Xiao B., Chen S., Qian Q., Liu Y. (2025). Predefined Attention-Focused Mechanism Using Center-Environment Features: A Machine Learning Study of Alloying Effects on the Stability of Nb_5_Si_3_ Alloys. Digit. Discov..

[38] Tang Y., Xiao B., Chen J., Chen S., Li Y., Liu F., Du W., Shen Y., Fan X., Qian Q. (2025). Machine Learning with Center-Environment Attention Mechanism for Multi-Component Nb Alloys. Trans. Nonferrous Met. Soc. China.

[39] Siddiqui A., Hine N.D.M. (2024). Machine-Learned Interatomic Potentials for Transition Metal Dichalcogenide Mo_1−x_W_x_S_2−2y_Se_2y_ Alloys. npj Comput. Mater..

[40] Liu Y., Mo Y. (2024). Assessing the Accuracy of Machine Learning Interatomic Potentials in Predicting the Elemental Orderings: A Case Study of Li-Al Alloys. Acta Mater..

[41] Ramakrishnan R., Dral P.O., Rupp M., von Lilienfeld O.A. (2014). Quantum Chemistry Structures and Properties of 134 Kilo Molecules. Sci. Data.

[42] Jain A., Ong S.P., Hautier G., Chen W., Richards W.D., Dacek S., Cholia S., Gunter D., Skinner D., Ceder G. (2013). Commentary: The Materials Project: A Materials Genome Approach to Accelerating Materials Innovation. APL Mater..

[43] Saal J.E., Kirklin S., Aykol M., Meredig B., Wolverton C. (2013). Materials Design and Discovery with High-Throughput Density Functional Theory: The Open Quantum Materials Database (OQMD). JOM.

[44] Tang Y., Xiao B., Chen J., Liu F., Du W., Guo J., Liu Y., Liu Y. (2023). Multi-Component Alloying Effects on the Stability and Mechanical Properties of Nb and Nb–Si Alloys: A First-Principles Study. Met. Mater. Trans. A.

[45] Guo J., Xiao B., Li Y., Zhai D., Tang Y., Du W., Liu Y. (2021). Machine Learning Aided First-Principles Studies of Structure Stability of Co_3_(Al, X) Doped with Transition Metal Elements. Comput. Mater. Sci..

[46] Horton M.K., Huck P., Yang R.X., Munro J.M., Dwaraknath S., Ganose A.M., Kingsbury R.S., Wen M., Shen J.X., Mathis T.S. (2025). Accelerated Data-Driven Materials Science with the Materials Project. Nat. Mater..

[47] Kresse G., Furthmüller J., Hafner J. (1994). Theory of the Crystal Structures of Selenium and Tellurium: The Effect of Generalized-Gradient Corrections to the Local-Density Approximation. Phys. Rev. B.

[48] Perdew J.P., Burke K., Ernzerhof M. (1996). Generalized Gradient Approximation Made Simple. Phys. Rev. Lett..

[49] Ko T.W., Ong S.P. (2023). Recent Advances and Outstanding Challenges for Machine Learning Interatomic Potentials. Nat. Comput. Sci..

[50] Jacobs R., Morgan D., Attarian S., Meng J., Shen C., Wu Z., Xie C.Y., Yang J.H., Artrith N., Blaiszik B. (2025). A Practical Guide to Machine Learning Interatomic Potentials—Status and Future. Curr. Opin. Solid State Mater. Sci..

[51] Yu H., Zhong Y., Hong L., Xu C., Ren W., Gong X., Xiang H. (2024). Spin-Dependent Graph Neural Network Potential for Magnetic Materials. Phys. Rev. B.

[52] Yu H., Liu B., Zhong Y., Hong L., Ji J., Xu C., Gong X., Xiang H. (2024). Physics-Informed Time-Reversal Equivariant Neural Network Potential for Magnetic Materials. Phys. Rev. B.

[53] Gilmer J., Schoenholz S.S., Riley P.F., Vinyals O., Dahl G.E. Neural Message Passing for Quantum Chemistry. Proceedings of the International Conference on Machine Learning, Sydney, NSW, Australia.

[54] Satorras V.G., Hoogeboom E., Welling M. E(n) Equivariant Graph Neural Networks. Proceedings of the International Conference on Machine Learning.

[55] Thomas N., Smidt T., Kearnes S., Yang L., Li L., Kohlhoff K., Riley P. (2018). Tensor Field Networks: Rotation- and Translation-Equivariant Neural Networks for 3D Point Clouds. arXiv.

[56] Fuchs F., Worrall D., Fischer V., Welling M. (2020). Se(3)-Transformers: 3d Roto-Translation Equivariant Attention Networks. Adv. Neural Inf. Process. Syst..

[57] Geiger M., Smidt T. (2022). E3nn: Euclidean Neural Networks. arXiv.

[58] Kresse G., Furthmüller J. (1996). Efficient Iterative Schemes for Ab Initio Total-Energy Calculations Using a Plane-Wave Basis Set. Phys. Rev. B.

[59] Kresse G., Furthmüller J. (1996). Efficiency of Ab-Initio Total Energy Calculations for Metals and Semiconductors Using a Plane-Wave Basis Set. Comput. Mater. Sci..

[60] Blöchl P.E. (1994). Projector Augmented-Wave Method. Phys. Rev. B.

[61] Kresse G., Joubert D. (1999). From Ultrasoft Pseudopotentials to the Projector Augmented-Wave Method. Phys. Rev. B.

