# Supplementary material for: Transfer Learning from Homogeneous to Heterogeneous: Fine-Tuning a Pretrained Interatomic Potential for Multicomponent Mo Alloys with Localized Substitutional Alloying
Source: Materials (Basel). 2026 Apr 23;19(9):1715. doi: 10.3390/ma19091715 (PMC13164484; doi:10.3390/ma19091715)
Supplement: Supplementary file 1 [file materials-19-01715-s001.zip › materials-4216004-supplementary.pdf]

Supplementary Materials of

Transfer Learning from Homogeneous to Heterogeneous: Fine-Tuning a Pretrained Interatomic Potential for Multicomponent Mo Alloys with Localized Substitutional Alloying

Lixin Fang [1,2], Liqin Qin [1,2], Limin Zhang [1,2], Hao Zhou [1,2], Xudong He [1,2], Zekun Ren [3,*], Tongyi Zhang [1,2,*] and Yi Liu [1,2,4,*]

1 Materials Genome Institute, Shanghai University, Shanghai 200444, China; fanglixin@shu.edu.cn (L.F.); bridgit-qin@shu.edu.cn (L.Q.); 16621672868@163.com (L.Z.); shuzhouhao@shu.edu.cn (H.Z.); hxd_yi@shu.edu.cn (X.H.)

2 State Key Laboratory of Materials for Advanced Nuclear Energy, Shanghai University, Shanghai 200444, China

3 Berkeley Education Alliance for Research in Singapore (BEARS), Create Tower, 1 Create Way, Singapore 138602, Singapore

4 Institute of Low-Dimensional Carbons and Device Physics, Shanghai University, Shanghai 200444, China

* Correspondence: danny.ren@bears-berkeley.sg (Z.R.); zhangty@shu.edu.cn (T.Z.); yiliu@shu.edu.cn (Y.L.)

Text S1 Architecture of MACE models

MACE adopts the pipeline as follows. Structure → Atomic Environment → embedding → (Interaction–Product–Update)^{K} → learnable features f_i → Readout → atomic energy E_i, as shown in Fig. 1. This architecture combines high-body-order ACE expansion with E(3)-equivariant message passing in an end-to-end framework[1–7]. Relative to conventional pairwise-message MPNNs, MACE improves expressivity through explicit higher-order interactions while maintaining a favorable accuracy–efficiency balance at shallow depth[2,5–7].

MACE adopts atomic-energy decomposition:

$$E = \sum_i E_i \tag{1}$$

Forces are obtained from energy gradients with respect to atomic coordinates:

$$\mathbf{F}_i = -\frac{\partial E}{\partial \mathbf{R}_i} \tag{2}$$

This single-energy formulation with auto-differentiated forces enforces consistency between energy and force predictions and supports stable convergence in joint training.

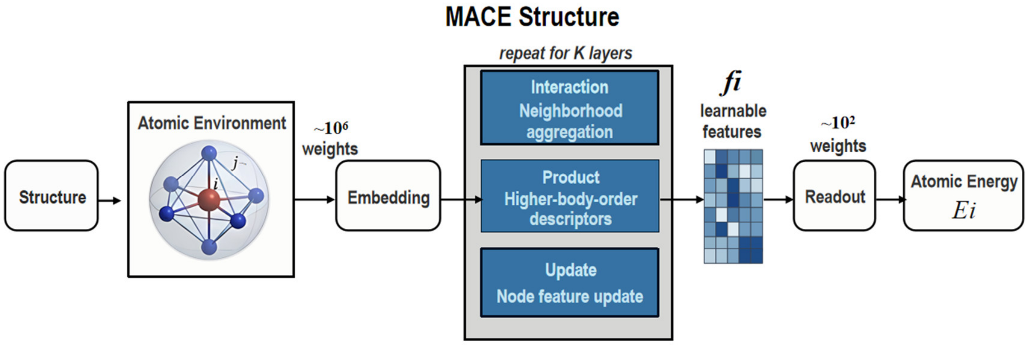

Figure S1. Schematic illustration of the MACE architecture. The pipeline follows Structure → Atomic Environment → embedding → (Interaction–Product–Update, repeated for K layers) → learnable atomic features f_i → Readout → atomic energy E_i.

The core characteristics in MACE are summarized as follows.

(1) Atomic Environment and embedding: The structure is mapped to a local neighborhood graph. Nodes encode element identities. Edge features are encoded with radial basis functions and spherical harmonics. Initial node states $h_i^{(0)}$ and edge representations are constructed under rotation/reflection equivariance c

onstraints, providing inputs for subsequent equivariant message construction. Th e corresponding node and edge representations can be written as:

$$\sigma_i^{(t)} = \left(r_i, z_i, h_i^{(t)}\right) \tag{3}$$

$$h_{i,k00}^{(0)} = W_{kz_i} \tag{4}$$

$$A_{i,klm}^{(1)} = \sum_{j \in \mathcal{N}(i)} R_{kl}^{(1)}\left(r_{ji}\right) Y_l^m\left(\widehat{r_{ji}}\right) W_{kz_j} \tag{5}$$

where ($\sigma_i^{(t)}$) denotes the node state tuple at layer (t), ($h_{i,k00}^{(0)}$) denotes the (k) -th invariant scalar channel of the initial node embedding, and ($A_{i,klm}^{(1)}$) denotes the first-layer equivariant Atomic Environment feature centered at atom (i) ,(i) is the center-atom index, (j) is a neighbor index, ($\mathcal{N}(i)$) is the neighbor set within the cutoff, (r_i) is the atomic coordinate, (z_i) is the chemical species la bel, ($h_i^{(t)}$) is the node feature at layer(t), (W_{kz}) is the learnable species-embed ding weight, ($r_{ji} = |r_j - r_i|$),($\widehat{r_{ji}} = \left(r_j - r_i\right)/r_{ji}$),($R_{kl}^{(1)}(\cdot)$) is the radial basis emb edding, and($Y_l^m(\cdot)$) is the spherical-harmonic angular basis.

(2) Feature Construction (Interaction–Product–Update): This stage is repeate d for K layers. Interaction aggregates neighborhood information. Product builds higher-order features through tensor products and symmetrization. Update writes messages back to nodes with residual updates, yielding $h_i^{(k+1)}$. The correlation order ν controls message body order (approximately $\nu + 1$) and governs the e xpressivity–cost trade-off.

(3) Learnable atomic features f_i: After K interaction layers, the model yiel ds atom-wise representations f_i for property prediction. Readout mainly operate s on invariant scalar channels ($0e$ components), ensuring rotational invariance o f predicted atomic energies.

(4) Readout: The Readout module maps f_i to atomic energy E_i. A hierarch ical aggregation scheme can combine information from different layers. Interme diate-layer readouts are typically linear; the final layer may use a lightweight MLP to improve nonlinear fitting capacity. This pipeline supports direct compa rison of zero-shot, scratch, and fine-tune paradigms in terms of energy/force err ors and transfer stability for doped systems.

Text S2 Computational parameters in the first-principles calculations

All first-principles calculations were carried out within the framework of density functional theory (DFT) using the Vienna Ab initio Simulation Package (VASP)[8,9]. The exchange-correlation functional was described by the generalized gradient approximation in the Perdew–Burke–Ernzerhof (PBE)[10,11] form. The interaction between ionic cores and valence electrons was treated using the projector augmented-wave (PAW)[12,13] method. A plane-wave cutoff energy of 500 eV was adopted for structural optimization and total-energy calculations.

For BCC and FCC Mo-based systems, Brillouin-zone sampling was performed using a Gamma-centered 5×5×5 k-point mesh. The electronic self-consistent convergence criterion was set to 1×10^{-5} eV, and the ionic relaxation proceeded until the residual force on each atom was below 0.02 eV/Å. Spin-polarized calculations were employed throughout to properly account for possible magnetic effects introduced by alloying elements. Considering the dilute substitution limit investigated in this work, structural relaxation was first carried out for the doped BCC-Mo and FCC-Mo configurations, followed by ionic relaxation with the cell volume and cell shape fixed.

Table S1 Overview of the ML models and their settings including training paradigms, data splitting, losses, and metrics in this work.

Module	Item	Setting
Training paradigm	Zero-shot	Direct inference (no parameter update)
Training paradigm	Scratch	Training from scratch
Training paradigm	Fine-tuning	Continued optimization from pretrained weights (full-parameter update)
Base model	Pretrained model	MACE-MP-0
Base model	Parameter scale	Small/medium/large models
Data setup	FT-Eq (equilibrium)	420 samples; 10-fold IID
Data setup	FT-NonEq (non-equilibrium)	6970 samples; 5-fold IID
Evaluation protocol	LODO-Strict (11 elements)	11 folds; per-fold test size 68
Evaluation protocol	LODO-Relaxed (11 elements)	11 folds; per-fold test size 8
Optimization target	Joint total loss	$L = \lambda_E L_E + \lambda_F L_F,\ \lambda_E = \lambda_F$
Metrics	Energy/force	MAE (meV/atom)/RMSE (meV/Å)
Hyperparameters	Energy key	REF_energy
	Forces key	REF_forces
	Learning rate	3×10^{-4}
	Weight decay	1×10^{-6}
	Batch size	8
	Validation batch size	8
	Max epochs	200
	Energy loss weight	30
	Force loss weight	30
	EMA	Enabled (ema_decay=0.99)
	Seed	42
	Default dtype	float32

References

(1) Drautz, R. Atomic Cluster Expansion for Accurate and Transferable Interatomic Potentials. *Phys. Rev. B* **2019**, *99* (1), 014104. https://doi.org/10.1103/PhysRevB.99.014104.

(2) Batatia, I.; Kovacs, D. P.; Simm, G.; Ortner, C.; Csányi, G. MACE: Higher Order Equivariant Message Passing Neural Networks for Fast and Accurate Force Fields. *Advances in neural information processing systems* **2022**, *35*, 11423–11436.

(3) Gilmer, J.; Schoenholz, S. S.; Riley, P. F.; Vinyals, O.; Dahl, G. E. Neural Message Passing for Quantum Chemistry. In *International conference on machine learning*; Pmlr, 2017; pp 1263–1272.

(4) Satorras, V. G.; Hoogeboom, E.; Welling, M. E (n) Equivariant Graph Neural Networks. In *International conference on machine learning*; PMLR, 2021; pp 9323–9332.

(5) Thomas, N.; Smidt, T.; Kearnes, S.; Yang, L.; Li, L.; Kohlhoff, K.; Riley, P. Tensor Field Networks: Rotation- and Translation-Equivariant Neural Networks for 3D Point Clouds. arXiv May 18, 2018. https://doi.org/10.48550/arXiv.1802.08219.

(6) Fuchs, F.; Worrall, D.; Fischer, V.; Welling, M. Se (3)-Transformers: 3d Roto-Translation Equivariant Attention Networks. *Advances in neural information processing systems* **2020**, *33*, 1970–1981.

(7) Geiger, M.; Smidt, T. E3nn: Euclidean Neural Networks. arXiv July 18, 2022. https://doi.org/10.48550/arXiv.2207.09453.

(8) Kresse, G.; Furthmüller, J. Efficient Iterative Schemes for Ab Initio Total-Energy Calculations Using a Plane-Wave Basis Set. *Phys. Rev. B* **1996**, *54* (16), 11169–11186. https://doi.org/10.1103/PhysRevB.54.11169.

(9) Kresse, G.; Furthmüller, J. Efficiency of Ab-Initio Total Energy Calculations for Metals and Semiconductors Using a Plane-Wave Basis Set. *Computational Materials Science* **1996**, *6* (1), 15–50. https://doi.org/10.1016/0927-0256(96)00008-0.

(10) Kresse, G.; Furthmüller, J.; Hafner, J. Theory of the Crystal Structures of Selenium and Tellurium: The Effect of Generalized-Gradient Corrections to the Local-Density Approximation. *Phys. Rev. B* **1994**, *50* (18), 13181–13185. https://doi.org/10.1103/PhysRevB.50.13181.

(11) Perdew, J. P.; Burke, K.; Ernzerhof, M. Generalized Gradient Approximation Made Simple. *Phys. Rev. Lett.* **1996**, *77* (18), 3865–3868. https://doi.org/10.1103/PhysRevLett.77.3865.

(12) Blöchl, P. E. Projector Augmented-Wave Method. *Phys. Rev. B* **1994**, *50* (24), 17953–17979. https://doi.org/10.1103/PhysRevB.50.17953.

(13) Kresse, G.; Joubert, D. From Ultrasoft Pseudopotentials to the Projector Augmented-Wave Method. *Phys. Rev. B* **1999**, *59* (3), 1758–1775. https://doi.org/10.1103/PhysRevB.59.1758.
